# Symmetric Dimethylarginine Is Not Associated with Cumulative Inflammatory Load or Classical Cardiovascular Risk Factors in Rheumatoid Arthritis: A 6-Year Follow-Up Study

**DOI:** 10.1155/2015/796562

**Published:** 2015-10-12

**Authors:** Theodoros Dimitroulas, James Hodson, Aamer Sandoo, Jacqueline P. Smith, Karen M. Douglas, George D. Kitas

**Affiliations:** ^1^Department of Rheumatology, Dudley Group of Hospitals NHS Trust, Russells Hall Hospital, Dudley DY1 2HQ, UK; ^2^Wolfson Computer Laboratory, University Hospital Birmingham NHS Foundation Trust, Queen Elizabeth Hospital Birmingham, Mindelsohn Way, Birmingham B15 2WB, UK; ^3^School of Sport, Health and Exercise Sciences, Bangor University, George Building, Bangor, Gwynedd, Wales LL57 2PZ, UK; ^4^Arthritis Research UK Epidemiology Unit, University of Manchester, Oxford Road, Manchester M13 9PT, UK

## Abstract

Symmetric dimethylarginine (SDMA) indirectly inhibits nitric oxide (NO) synthesis and predicts cardiovascular and all-cause mortality in high-risk patients. The aim of our study was to investigate the associations of cumulative inflammatory burden (assessed by serial measurements of inflammatory markers) and classical cardiovascular (CV) disease risk factors with SDMA in RA patients. 201 RA patients (155 females, median age 67 (59–73)) were assessed at baseline (2006). Classical CV disease risk factors were recorded and systemic inflammation was determined by the measurement of C-reactive protein (CRP) and erythrocyte sedimentation rate (ESR). At follow-up (2012) SDMA levels were measured by enzyme-linked immunosorbent assay. Mean SDMA levels in RA population were 0.40 (0.40–0.53) *μ*mol/L. No significant association between SDMA and cumulative inflammatory load was established in the analysis. SDMA levels were not found to be significantly related to CV disease risk factors. We explored the potential relationship between SDMA and cumulative inflammatory burden in patients with RA and obtained negative results. SDMA did not relate to CV disease risk factors in our population and its clinical significance as a surrogate marker of endothelial dysfunction in patients with RA remains to be determined.

## 1. Introduction

There is a substantial amount of evidence suggesting that cardiovascular (CV) disease is the most common cause of premature mortality in patients with rheumatoid arthritis (RA) [1]. Even after adjusting for traditional CV risk factors, the magnitude of CV risk in RA is increased by approximately 50% compared to the general population [2]. Components of cardiometabolic syndrome such as dyslipidemia, insulin resistance, and hypertension are more common in patients with RA leading to endothelial dysfunction and coronary artery disease [3].

It is well-recognised that the endothelium is the key regulator of vascular function by controlling vascular tone, smooth muscle proliferation, platelet aggregation, and migration of adhesion molecules [4]. Inflammation in RA has a systemic nature and inflammatory molecules and cytokines exhibit deleterious effects on the vasculature resulting in endothelial dysfunction. Derangement of endothelial homeostasis is an early preclinical marker of atherosclerosis and several studies have demonstrated that endothelial dysfunction is more prevalent in RA patients compared to controls [5]. The damaged endothelium disrupts the metabolism of nitric oxide (NO) which is the most important vasoactive agent for the maintenance of basal vasodilator tone and vascular patency. Therefore, dysregulation of NO production has been considered as the cornerstone of endothelial dysfunction and much attention has been paid to the identification of pathways associated with suppression of NO synthase activity, the enzyme responsible for NO synthesis.

Dimethylarginines are by-products of the posttranslational methylation of arginine residues in proteins and they interfere with NO formation by inhibiting NO synthase which, in turn, leads to endothelial dysfunction. Asymmetrical dimethylarginine (ADMA) is the most potent inhibitor of NO synthase and has emerged as a novel biomarker of CV outcome and mortality among patients with intermittent and high CV risk, as well as in the general population [6, 7]. Since the details of the mechanisms accompanying this effect are unravelling, interest has started to divert towards symmetrical dimethylarginine (SDMA), the inactive congener of ADMA which has not been studied to a similar extent. SDMA is mainly excreted by kidneys and may provide a mechanistic link between renal dysfunction and CV disease. Elevated SDMA levels are associated with adverse outcomes in stroke [8], worse prognosis in patients referred for coronary angiography [9], and all-cause and CV mortality in the general population [10].

These findings suggest that SDMA may have an independent role in the pathogenesis of endothelial dysfunction and CV disease, by inhibiting both renal and cellular uptake of L-arginine, thus also potentially diminishing NO bioavailability [11, 12]. In addition, SDMA induces monocyte-mediated reactive oxygen species production [13] and has been described as a proinflammatory agent in chronic kidney disease [14]. In RA, the magnitude of the inflammatory response over time (cumulative inflammatory burden) rather than a single determination of inflammatory markers may be more important for the development and progression of vascular injury [15]. Despite emerging data in several conditions associated with atherosclerosis and CV disease, the role of SDMA in RA remains largely unexplored. The aim of the present study was to investigate whether cumulative systemic inflammatory burden and/or classical CV risk factors in RA associate with SDMA in a large well-characterized cohort of patients with long-standing RA.

## 2. Materials and Methods

### 2.1. Study Participants

We studied 201 patients with RA. The patients were part of the Dudley Rheumatoid Arthritis Comorbidity Cohort (DRACCO), a prospective study examining CV burden in RA. These individuals have participated in other studies of cardiovascular risk factors, and patient characteristics and detailed methods have been previously described [16, 17]. In summary, 201 out of 400 patients initially recruited in 2006 performed a follow-up visit in 2012. Seventy-eight patients had died since 2006 and the remaining 121 declined to participate for personal reasons. Exclusion criteria for the present study were confirmed acute coronary syndrome, evidence of chronic kidney disease, or serious psychiatric disorders according to their medical records. All patients met the 1987 revised RA criteria of the American College of Rheumatology [18]. The study received ethics approval from The Black Country Research Ethics Committee. All participants gave their written informed consent according to the Declaration of Helsinki.

### 2.2. Protocol for Baseline

Patients were evaluated after a 12-hour overnight fast and underwent a standardised clinical interview, physical examination, and detailed review of their medical history and hospital records. Height and weight were measured and the body mass index (BMI) was calculated (using a TANITA Body Composition Analyser BC-418). Disease activity score (DAS28) [19] and physical function using the Health Assessment Questionnaire (HAQ) [20] were recorded. Chart review with RA treatment and current therapy for other indications was performed. A blood sample was also obtained on the same day for the assessments of routine hematologic and biochemistry, lipid profile, fasting glucose, fasting insulin, and acute phase response. Insulin resistance was determined by using the Homeostasis Model Assessment Insulin Resistance (HOMA-IR) and Quantitative Insulin Sensitivity Check Index (QUICKI), as previously described [21, 22]. All biochemical tests were carried out in the Biochemistry Laboratory at Russells Hall Hospital, The Dudley Group NHS Foundation Trust, UK.

### 2.3. Protocol for Follow-Up Visit

Patients reported to the same clinical research facility as in the baseline assessment in 2012 after a 12-hour overnight fast. A venous blood sample was collected to assess SDMA levels. The SDMA assay is based on the method of competitive enzyme-linked immunoassays. The sample preparation includes the addition of a derivatization-reagent for SDMA coupling. During the incubation period, the target SDMA in the sample competes with the SDMA derivative (tracer) immobilised on the wall of the microtiter plates for the binding of the polyclonal antibodies. The SDMA in the sample displaces the antibodies out of the binding to the tracer. Therefore, the concentration of the tracer-bound antibody is inversely proportional to the SDMA concentration in the sample. The absorbance is measured at 450 nm and patient samples are read from a standard curve.

The intra-assay standard deviation was 7.5% and interassay was 6%. The lowest amount detected was 0.05 *μ*mol/L.

### 2.4. Cumulative Inflammatory Burden

Detailed information on erythrocyte sedimentation rate (ESR) and C-reactive protein (CRP) from the baseline visit to the follow-up visit for each patient was used to calculate cumulative inflammatory burden. A quarterly measurement of CRP and ESR for each year the patient was in the study was used to calculate the area under the curve (AUC) for each parameter. ESR was measured using the StaRRsed Auto Compact blood sedimentation instrument (Mechatronics, Zwaag, Netherlands) and CRP was measured using the VITROS 5,1 FS Chemistry System (Ortho-Clinical Diagnostics, Rochester, NY, USA).

### 2.5. Statistical Analysis

Several of the variables being considered, including SDMA, followed skewed distributions. Where this was the case, log_2_-transformations were applied to the data prior to the analysis, to allow the use of parametric tests. AUCs for ESR and CRP data were calculated for the quarterly measurements made between 2006 and 2012. Since some patients had missing data at the start or end of the period, the AUCs were dependent on the length of time the data were available. For this reason, the resulting AUCs were divided by the total period that data were available for each patient to produce average AUCs (AAUCs).

The log_2_-transformed SDMA values were then compared across categorical factors using independent samples *t*-tests for dichotomous factors and one-way ANOVA where there were multiple categories. For the continuous factors, regression models were produced to quantify relationships with SDMA. All of the factors from the univariable analysis were then considered for inclusion in a multivariable regression model, using a forward stepwise entry method, in order to account for the potential effects of confounding.

All analyses were performed using IBM SPSS Statistics 22 (IBM Corp. Armonk, NY). Missing data was excluded on a per analysis basis, and *p* < 0.05 was deemed to be indicative of statistical significance.

## 3. Results and Discussion

### 3.1. Results

The characteristics of the participants at baseline and follow-up as well as the general demographics are summarised in Table 1. The majority of the patients were female with relatively low disease activity score and acute phase response, parameters indicating optimal RA control despite long disease duration.

Data were available for 201 patients, of whom 197 had SDMA measurements. After being log_2_-transformed, SDMA was found to closely follow a normal distribution, with the exception of 11 (6%) patients with SDMA values >1. These outliers were excluded from the data, in order to make parametric analysis valid and to prevent them from becoming excessively influential in the tests performed.

**Figure 1:**
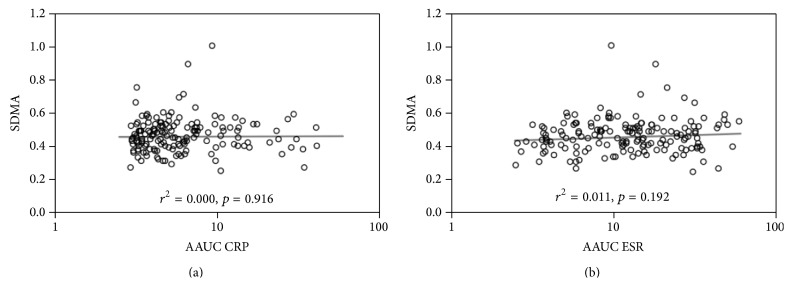
Graphic demonstration of the associations between SDMA and AAUCs of CRP (a) and ESR (b). Cumulative inflammation is plotted on a logarithmic scale, and the fit line and related statistics are based on the regression models in Table 2. SDMA: symmetric dimethylarginine, ESR: erythrocyte sedimentation rate, CRP: C-reactive protein, and AAUC: average area under the curve.

Univariable analysis of SDMA found only two factors to be significant predictors, namely, estimated glomerular filtration rate (eGFR) (*p* = 0.024) and creatinine (*p* = 0.016) (Table 2). For eGFR, the relationship was negative, with a one unit increase associated with a 0.3% (95% CI: 0.0%, 0.5%) reduction in SDMA. Creatinine, on the other hand, had a positive relationship with SDMA, with a twofold increase in the former associated with a 12.0% (95% CI: 2.2%, 22.8%) increase in the latter. Neither of the cumulative inflammatory markers was found to be significantly correlated with SDMA, with *p* = 0.916 for the AAUC of CRP and *p* = 0.192 for the AAUC of ESR (Table 2). This is demonstrated graphically in Figure 1.

Univariable analysis of categorical factors found no significant associations between SDMA, traditional CV disease risk factors, and treatment with steroids, methotrexate, tumour necrosis factor-alpha inhibitors, or antihypertensive agents (Table 3).

To consider the potential for confounding factors, multivariable analyses were performed, using forward stepwise regression models, with all of the variables in Tables 2 and 3 as potential covariates. Creatinine was the only factor entered into the final model, which was expected, since none of the other factors were significant in univariable analysis with the exception of eGFR, which was highly correlated with creatinine (Pearson's *r*: −0.83, *p* < 0.001).

### 3.2. Discussion

In this study, we assessed for the first time the relationship between SDMA and cumulative inflammatory burden and traditional CV risk factors in RA patients. Although SDMA has been reported as a surrogate marker of endothelial dysfunction in a number of conditions characterised by excess CV morbidity, no significant associations with disease-related inflammation, metabolic factors, and CV risk scores were detected in our RA population.

RA is characterized by a heightened inflammatory state and the chronic intense interaction occurring between vasculature and inflammation could promote vascular changes conducive to increased CV risk. Thus, it is not surprising that morphological markers of atherosclerosis such as intima-media thickness have been correlated with the magnitude of systemic inflammation assessed by higher CRP values [23] and patients with elevated ESR are at increased risk of CV death compared to those with normal levels [24]. However, recent insights argue against a direct impact of the inflammatory process on vascular wall, demonstrating that systemic markers of inflammation do not correlate with changes in noninvasive vascular assessments of microvascular and macrovascular endothelial function characterizing different stages of atherosclerosis [4].

On the other hand, reports on the association between soluble biomarkers of endothelial dysfunction and indices of systemic inflammation have provided inconsistent results [25, 26]. Particularly for the dimethylarginines, conflicting associations between ADMA and the acute phase response have been reported, with some studies establishing positive correlations with CRP and ESR [17, 27] and others yielding negative results [28, 29]. These discrepancies can be attributed to different methodological approaches, cross-sectional study designs, inclusion of small and heterogeneous patient populations, and variations in disease duration, activity, and treatment. Importantly, the method of characterising the effect of inflammation on the vasculature may also influence the findings of studies. For example, the magnitude and chronicity of inflammation assessed with historical measures of cumulative inflammatory burden probably represents a more reliable marker of the total inflammatory burden to which an RA individual has been exposed during the course of the disease than a single reading of CRP or ESR [15]. To lend more support to this, cumulative inflammation shows better associations with noninvasive assessments of peripheral vascular function and morphology when compared with current inflammatory levels [5]; however, only a small number of studies have employed such an approach. Positive correlations between cumulative inflammatory burden and arterial stiffness have been reported in RA [30] and we recently found similar associations for ADMA [17].

Despite previous observations, we did not demonstrate any correlation between SDMA and cumulative inflammatory burden. One reason may be the effective control of inflammatory disease in our cohort. Other potential explanations include differences in biology between ADMA and SDMA. It also appears that dimethylarginines may promote endothelial dysfunction through different mechanisms even beyond NO synthase inhibition [31]. ADMA mediates oxidative stress in vasculature through endothelial NO synthase uncoupling and vice versa. Inflammation influences its metabolic pathways, resulting in elevated levels of ADMA, predominantly due to dimethylarginine dimethylaminohydrolase inhibition [32]. Inflammation and oxidative pathways represent important mechanisms accounting for the pathogenetic role of ADMA in CV disease. However, there has been limited understanding regarding the interplay between SDMA and inflammation. It is worth noting that SDMA remained unchanged in patients with acute bacterial infection during the course of the disease whilst changes in ADMA levels were noticed during the recovery phase of the infection [33].

SDMA has been associated with the expression of proinflammatory cytokines in patients with chronic kidney disease [14], but the aetiology of chronic inflammatory status characterising the uremic conditions differs from that of high-grade systemic inflammatory diseases such as RA. SDMA may exhibit proinflammatory properties associated with abnormal renal function contributing to the moderate inflammation present in the majority of uremic patients and corroborating the previously demonstrated increase in monocyte-mediated reactive oxygen species production [13]. However, the lack of association with inflammation in other populations may indicate its main biologic role as a uremic toxin and marker of renal function. In that respect, our results confirm the presence of a relationship between serum creatinine and SDMA in keeping with the findings from a meta-analysis by Kielstein et al. [34].

Traditional CV risk factors are more prevalent and poorly controlled in RA compared to general population, but they only partially account for the development of atherosclerosis and increased CV risk [35]. It has been hypothesized that such risk factors operate differently in RA and general population, as some studies have reported that systemic inflammation provides positive modulation to the effects of the established factors in the vasculature and CV disease [36]. To lend more support, the appreciation that risk algorithms used for CV risk stratification in the general population underestimate the risk of future events in RA patients underlines the complexity of interrelations between traditional and disease-related risk factors in this condition [37]. Metabolic alterations such as insulin resistance, obesity, and aberrant lipid metabolism are more frequent in patients with RA and are associated with increased disease activity [38]. The association between cardiometabolic syndrome and RA may be reciprocal, with chronic low-grade inflammation characterising insulin resistance and high-grade RA-related systemic inflammatory activity reinforcing each other, resulting in a vicious circle promoting oxidative stress and vascular damage. In our cohort, CV risk factors were not found to influence SDMA levels and, to the best of our knowledge, this is the first study to investigate such associations, not only in RA, but also in other high-risk patient populations. Similar results have also been reported for ADMA in patients with RA [17], coronary artery [39], and cerebrovascular disease [40]. Whether SDMA represents a surrogate marker of endothelial dysfunction in RA remains to be determined. However, the lack of association with cumulative inflammatory burden and classic CV factors may suggest that, apart from a reliable indicator of renal function, its utility as mediator of vascular injury in RA may be limited.

Although the last decades have witnessed significant advances in treatment of RA, the mortality gap between RA individuals and general population has been widening, mainly due to CV disease [41]. Subsequently, CV risk prevention should constitute a key goal in management of this condition [42]. Besides clinical recommendations [43], there is an urgent need in identifying biomarkers to allow the early recognition of RA patients at increased risk for CV events and to facilitate individualised treatment strategies. Dimethylarginines have been shown to predict CV mortality and morbidity in the general [7, 44] population, but it is questionable whether these observations are applicable to RA where the pathogenesis of vascular damage may arise through different mechanisms triggered by inflammation and immune activation. It is, therefore, unlikely that a single biomarker could encompass the various processes involved in endothelial dysfunction in RA. However, disruption of NO metabolism is crucial, with ADMA and SDMA mediating, probably through different pathways, the deleterious effects of inflammation on the vascular wall and promoting endothelial injury and atherosclerosis. Such a process may be more pronounced in chronic high-grade inflammatory conditions such as RA, suggesting an important role for these molecules, whose potential to predict future CV events in RA patients remains to be investigated in large prospective studies with hard CV disease end points.

Our study has several strengths, as we investigated the association between SDMA and cumulative inflammation in a large real-life RA population with sufficient assessment and measurement of classical CV risk factors at baseline. Furthermore, repeated measurement of inflammatory markers made it possible to take variability in disease activity into account. In contrast, low disease activity in our patients may have contributed to the negative results. The absence of serial measurements of SDMA is another limitation, but we are not aware of any data suggesting fluctuation of SDMA levels over time. In addition, we excluded outliers with extremely high SDMA from the analysis to ensure the validity of our models. Finally, we did not find any associations between SDMA and antirheumatic treatment. However, large longitudinal studies with serial measurements of ADMA and/or SDMA are warranted to address the ability of dimethylarginines as indicators of potential endothelial function improvement following treatment with synthetic and biologic disease modifying drugs. Until today, only a small study showed reduction in ADMA levels achieved with biologic drugs [45] whilst others failed to demonstrate any changes in concentrations of ADMA and SDMA in RA and ankylosing spondylitis [46, 47].

## 4. Conclusion

In summary, our findings suggest that SDMA is independent of cumulative inflammatory load assessed by quarterly measurement of CRP and ESR for each year, as well as established CV risk factors in RA, but associations between SDMA and proinflammatory cytokines cannot be categorically excluded. Altered NO homeostasis is considered an important mechanism for vascular changes in RA and previous observations have revealed that reduced NO availability is associated with inflammation. However, the specific role of SDMA in the development of atherosclerosis and abnormal endothelial function in this population warrants further investigations.

## Figures and Tables

**Table 1:** Patient characteristics at baseline and at follow-up (Sandoo A, Dimitroulas T, Hodson J, Smith JP, Douglas KM, Kitas GD. Cumulative inflammation associates with asymmetric dimethylarginine in rheumatoid arthritis: a 6 year follow-up study. Rheumatology. 2014 Sep 3. [Ahead of print]. Reproduced with permission from Oxford University Press).

	Baseline (2006)	Follow-up (2012)
General characteristics		
Age (years)	61 (53–67)	67 (59–73)
Sex female *N* (%)	155 (77%)	155 (77%)
Body mass index (kg/M^2^)	27 (24–30)	28 (24–32)
Disease characteristics		
Disease duration (years)	10 (4–18)	16 (11–25)
Rheumatoid factor positive *N* (%)	148 (74%)	148 (74%)
Anti-CCP positive *N* (%)	123 (61%)	123 (61%)
DAS28	4.0 (3.1–4.8)	3.1 (2.5–4.0)
HAQ	1.3 ± 0.9	1.6 ± 0.9
C-reactive protein (mg/L)	7.5 (4.3–16)	3 (2.9–8.5)
Erythrocyte sedimentation rate (mm/hr)	17 (8–30)	12 (5–23)
SDMA (*μ*mol/L)	—	0.47 (0.40–0.53)
Cardiovascular disease risk factors		
Hypertension *N* (%)	132 (66%)	130 (65%)
Dyslipidemia *N* (%)	115 (57%)	158 (79%)
Insulin resistance *N* (%)	65 (32%)	53 (26%)
Diabetes *N* (%)	7 (4%)	21 (10%)
Global CVD risk scores		
Framingham Risk Score (%)	4 (1–8)	8 (5–13)
Reynolds Risk Score (%)	6 (3–13)	8 (3–14)
RA Medications		
Methotrexate *N* (%)	128 (64%)	122 (61%)
Prednisolone *N* (%)	58 (29%)	51 (25%)
NSAID's *N* (%)	47 (23%)	26 (13%)
Cyclooxygenase II inhibitors *N* (%)	14 (7%)	5 (3%)
Anti-TNF*α* therapy *N* (%)	20 (10%)	57 (28%)
Tocilizumab *N* (%)	—	3 (2%)
Cardiovascular medications		
Antihypertensive *N* (%)	81 (40%)	79 (39%)
Antihypercholesterolemic *N* (%)	33 (16%)	74 (37%)
Beta-blocker *N* (%)	32 (16%)	22 (11%)
Calcium channel blocker *N* (%)	26 (13%)	27 (13%)

Results are expressed as median (25th to 75th percentile values), mean ± standard deviation or number (percentage). DAS28 = disease activity score in 28 joints. Anti-TNF*α* = antitumour necrosis factor alpha; CCP = citrullinated protein antibody; HAQ = Health Assessment Questionnaire; NSAID's = nonsteroidal anti-inflammatory drugs; SDMA: symmetric dimethylarginine.

**Table 2:** Univariable analysis of continuous factors.

	SDMA	
	Coefficient (95% CI)	*p* value
BMI	−0.5% (−1.1%, 0.0%)	0.063
HR	−0.1% (−0.3%, 0.1%)	0.380
SBP	0.0% (−0.1%, 0.2%)	0.653
DBP	0.1% (−0.2%, 0.3%)	0.633
Log_2_ Reynolds Risk Score	1.5% (−0.8%, 3.9%)	0.191
Log_2_ Framingham Risk Score	−0.1% (−3.2%, 3.2%)	0.955
eGFR	−0.3% (−0.5%, 0.0%)	0.024^*∗*^
Log_2_ creatinine	12.0% (2.2%, 22.8%)	0.016^*∗*^
Log_2_ AAUC CRP	0.2% (−3.4%, 3.9%)	0.916
Log_2_ AAUC ESR	2.0% (−1.0%, 5.0%)	0.192

Results from univariable regression models, with log_2_-transformed dependent variables. Coefficients were then antilogged, to represent the percentage change in the untransformed outcome for a unit increase in the factor. For log_2_-transformed factors, the coefficient represents the increase in the outcome for a twofold increase in the untransformed factor. ^*∗*^Significant at *p* < 0.05. BMI: body mass index (kg/M^2^), HR: heart rate, SBP: systolic blood pressure, DBP: diastolic blood pressure, eGFR: estimated glomerular filtration rate, CRP: C-reactive protein, and ESR: estimated sedimentation rate.

**Table 3:** Univariable analysis of categorical factors.

	*N*	SDMA	
		Average	*p* value
High cholesterol			0.275
No	122	0.45 (0.44–0.47)	
Yes	64	0.47 (0.44–0.50)	
Hypertension			0.866
No	69	0.46 (0.44–0.48)	
Yes	117	0.46 (0.44–0.47)	
Insulin resistance			0.149
No	136	0.46 (0.45–0.48)	
Yes	50	0.44 (0.42–0.47)	
Smoking			0.957
Never	84	0.46 (0.44–0.48)	
Previously	78	0.45 (0.43–0.48)	
Currently	22	0.46 (0.42–0.50)	
Family history of cardiac events			0.506
No	102	0.46 (0.44–0.48)	
Yes	84	0.45 (0.43–0.47)	
MTX			0.837
No	69	0.46 (0.43–0.48)	
Yes	117	0.46 (0.44–0.48)	
Steroids			0.239
No	140	0.45 (0.44–0.47)	
Yes	46	0.47 (0.44–0.51)	
Anti-TNF			0.599
No	133	0.46 (0.44–0.47)	
Yes	53	0.46 (0.44–0.49)	
Antihypertensive			0.463
No	116	0.45 (0.44–0.47)	
Yes	70	0.47 (0.44–0.49)	

Data reported as “geometric mean (95% confidence interval)”, with *p* values from independent samples *t*-tests/one-way ANOVA on log_2_-transformed values. Significant at *p* < 0.05.
